# Production of Oxidation-Resistant Cu-Based Nanoparticles by Wire Explosion

**DOI:** 10.1038/srep18333

**Published:** 2015-12-16

**Authors:** Go Kawamura, Samuel Alvarez, Ian E. Stewart, Matthew Catenacci, Zuofeng Chen, Yoon-Cheol Ha

**Affiliations:** 1 Duke University, Department of Chemistry, Durham, North Carolina 27708, United States; 2 Toyohashi University of Technology, Department of Electrical and Electronic Information Engineering, 1-1 Hibarigaoka, Tempaku-cho, Toyohashi, Aichi 441-8580, Japan; 3 Tongji University, Department of Chemistry, Shanghai 200092, PR China; 4 Korea Electrotechnology Research Institute, Creative and Fundamental Research Division, Changwon 642-120, Korea

## Abstract

The low performance or high cost of commercially available conductive inks limits the advancement of printed electronics. This article studies the explosion of metal wires in aqueous solutions as a simple, low-cost, and environmentally friendly method to prepare metallic nanoparticles consisting of Cu and Cu alloys for use in affordable, highly conductive inks. Addition of 0.2 M ascorbic acid to an aqueous explosion medium prevented the formation of Cu_2_O shells around Cu nanoparticles, and allowed for the printing of conductive lines directly from these nanoparticles with no post-treatment. Cu alloy nanoparticles were generated from metal wires that were alloyed as purchased, or from two wires of different metals that were twisted together. Cu nanoparticles alloyed with 1% Sn, 5% Ag, 5% Ni and 30% Ni had electrical conductivities similar to Cu but unlike Cu, remained conductive after 24 hrs at 85 °C and 85% RH.

Printed electronics has the potential to enable rapid prototyping and low-cost production of functional circuits. Achieving this vision requires the development of low-cost inks that can deliver the desired level of performance and environmental stability with minimal post-processing. Current conductive inks based on organometallic precursors or metal nanoparticles (NPs) exhibit near-bulk-metal conductivity (1.0 × 10^−7^ Ω m)^1^,^2^. This high conductivity is usually achieved only after heat treatments at temperatures greater than 200 °C under reducing conditions to sinter and connect NPs^3^,^4^. These high post-processing temperatures limit the number of compatible substrates or processing systems^5^,^6^,^7^,^8^. Current conductive inks also rely on the use of Au and Ag due to the high conductivity of these metals, and their stability in air and water. However, the reliance on rare noble metals makes conductive inks fairly expensive, a problem which will only get worse as the use of these inks becomes more common. Conductive polymers require little post processing and are potentially a sustainable alternative to noble metal-based inks, but these materials are much less conductive (>10^−2^ Ω m)^9^,^10^.

Cu NPs are a promising replacement for Au and Ag NPs because Cu is 100 times cheaper ($7 kg^−1^) than Ag and possesses a conductivity (1.68 × 10^−8^ Ω m) between Au (2.44 × 10^−8^ Ω m) and Ag (1.59 × 10^−8^ Ω m). The main problem with Cu NPs is their sensitivity to oxygen^11^,^12^. Cu oxides on the surface of NPs are usually removed by sintering at temperatures greater than 200 °C in a reducing atmosphere (e.g. H_2_), and the oxidation of Cu after sintering reduces the electrical conductivity of the final device. Attempts to solve the oxidation problem include coating Cu NPs with graphene^13^, poly(N-vinylpyrrolidone) (PVP)^14^,^15^,^16^, and Ag^17^. However, graphene increases the contact resistance between NPs, PVP requires high sintering temperatures for removal, and the deposition of Ag adds cost.

Cu alloy NPs could be a viable alternative to Cu NPs coated with protective layers. NP’s of several copper alloys have been produced to date, including Cu-Au, Cu-Ag, Cu-Ni, Cu-Al, and Cu-Zn^18^,^19^,^20^,^21^,^22^,^23^. However, many of the preparation procedures require toxic chemicals, and their detailed corrosion resistance has yet to be reported.

Wire explosion (WE) is one of the simplest, cost effective, versatile, and environmentally friendly methods for the production of metal NPs. It is also easy to scale up while retaining the quality of NPs. Instantaneous evaporation of macroscopic metal wires is only performed by huge electric current to form nanoparticles. Thus WE consumes only metal wires and electricity, and generates little waste^24^,^25^. More than 20 different metal-based NPs, including Cu and several of its alloys have been prepared by WE in various gaseous and liquid environments^26^,^27^,^28^,^29^,^30^,^31^,^32^,^33^,^34^,^35^,^36^,^37^. Disadvantages of WE relative to chemical methods include the fact that it is more difficult to produce a NP product that is monodisperse in size. Although this may not be a problem for the use of these NPs in conductive inks, the NPs can be classified by sedimentation or centrifugation if required. To date, there have been no studies on the electrical properties and corrosion resistance of Cu alloy NPs produced by WE.

This work reports the preparation of several novel Cu alloy NPs with WE, including 99Cu-1Sn (1Sn), 95Cu-5Ag (5Ag), 95Cu-5Ni (5Ni), 70Cu-30Ni (30Ni) and 70Cu-30Zn (30Zn). NPs were prepared from metal wires that were either alloyed as purchased or from two separate wires that were twisted together. The composition of the NPs could be changed simply by twisting different numbers of wires together with different compositions. Wires were exploded in water with ascorbic acid added as a mild reducing agent to decrease the oxidation of Cu. This method to produce NPs generates no waste except for the nontoxic aqueous solution of ascorbic acid. Films of as-prepared NPs exhibited electrical resistivities as low as 5.25 × 10^−5^ Ω m after drying in air at room temperature. The resistivity could be lowered further to 1.64 × 10^−6^ Ω m by sintering at 200 °C in H_2_. Conductive lines drawn using Cu alloy NPs were more resistant to corrosion than Cu NPs at high temperature and under humid conditions.

## Results and Discussion

### Cu NPs prepared by WE

**Figure 1:**
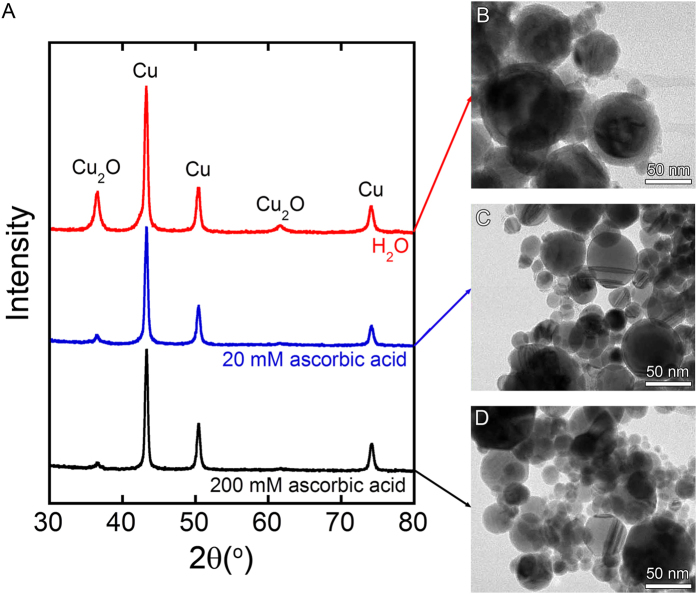
(**A**) XRD patterns of samples prepared by WE of Cu wires in deionized water and water containing 20 and 200 mM ascorbic acid. (**B**–**D**) show the corresponding TEM images.

As shown by the XRD pattern in Fig. 1, the NPs prepared by WE of Cu wires in deionized water are composed of a mixture of Cu and Cu_2_O (Fig. 1A red). The TEM image shows the formation of core-shell Cu@Cu_2_O NPs with diameters less than 100 nm, and shells less than 12 nm in thickness (Fig. 1B). When the sample was prepared in an aqueous solution containing 20 mM ascorbic acid, much weaker Cu_2_O peaks were observed in the XRD pattern (Fig. 1A blue), and the TEM image showed the thickness of the Cu_2_O shell was <1 nm (Fig. 1C). Increasing the ascorbic acid concentration to 200 mM further suppressed the oxidation of Cu (Fig. 1A black and Fig. 1D). Ascorbic acid is sometimes used for reducing Cu ions to form Cu nanoparticles^38^,^39^,^40^,^41^. Required energy to reduce Cu ions by ascorbic acid is generated as heat by the explosion of wire in this work.

**Figure 2:**
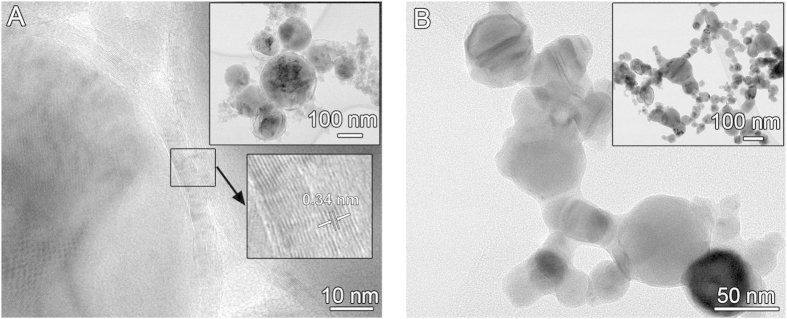
TEM images of NPs prepared by WE. (**A**) Cu NPs produced by explosion of Cu wire in ethanol are coated by graphitic carbon with an interlayer spacing of 0.34 nm. (**B**) Cu NPs exploded in N_2_ gas are aggregated into clusters.

Previous work has studied the suppression of oxide formation during WE through the use of organic solvents or inert gases^27^. However, we found that the explosion of Cu NPs in organic solvents resulted in the formation of graphitic carbon on the surface of the NPs, presumably because of a large amount of carbon element in the solvent (Fig. 2A). These carbon layers will increase the contact resistance between NPs. For example, Cu NPs coated with graphitic carbon were previously prepared in a reducing flame synthesis, and the inkjet-printed lines displayed a relatively high electrical resistivity of 1 × 10^−2^ Ω m^13^. Metal NPs prepared by WE in inert gas should be free of carbonaceous surface layers, but NPs prepared under gaseous conditions tend to aggregate (Fig. 2B). Due to this aggregation, the conductive lines drawn with the NPs are porous, and thus have low electrical conductivity^42^. The conductivity of the lines did not improve substantially after sintering at 200 °C in H_2_ for 30 min. This is because higher temperatures are needed to reduce the porosity significantly.

Surprisingly, there has been no previous attempt to prevent the oxidation of metal NPs exploded in water with a reducing agent. The fact that adding a reducing agent to the explosion medium does help to prevent the oxidation of the NPs points to the relatively unexplored potential of using chemical methods to control the surface chemistry of NPs produced with WE. Although we have not surveyed what other reducing agents can be used in this concept, agents with low molecular weights should be employed, eliminating the possibility to form carbonaceous surface layers or to get contaminated.

After drying at room temperature in air, the typical resistivity of the lines of Cu NPs produced with 200 mM ascorbic acid was 8.25 × 10^−5^ Ω m. This resistivity decreased to 1.72 × 10^−6^ Ω m after sintering at 200 °C in H_2_ for 30 min. A much higher resistivity of 1.61 × 10^−2^ Ω m was observed for the Cu NPs prepared using the 20 mM ascorbic acid solution. This is presumably because the thin Cu_2_O shell (<1 nm in Fig. 1C) obstructed the conduction of electrons between Cu NPs. Sintering helped to reduce the Cu_2_O shell, and decreased the resistivity to 2.38 × 10^−5^ Ω m. In contrast, conductive lines drawn from the Cu-Cu_2_O NPs prepared by WE in water without added ascorbic acid were not conductive before or after sintering. These results are summarized in Table 1. Clearly, the addition of ascorbic acid was critical to minimizing the resistivity of the metal lines.

In addition to the effect of surface oxide, we examined the effect of a polymeric stabilizer and particle size on the electrical resistivity of metal lines made from Cu NPs. To examine the effect of adding a polymeric stabilizer, copper wires were exploded in an aqueous solution containing 0.16 mM PVP (molecular weight = 10^4^) and 200 mM ascorbic acid. The resulting lines exhibited a resistivity 7 times higher (5.60 × 10^−4^ Ω m) than the samples without PVP (8.25 × 10^−5^ Ω m), although there was not an observable difference in the size or dispersion of the NPs. Lines drawn with 200 mM ascorbic acid solution containing 40 vol% of commercially available Cu alloy micro particles (50 μm brass and 150 μm bronze, Alfa Aesar) exhibited no electrical conductivity, even after sintering at 200 °C under H_2_ for 30 min. In both cases, the high resistivity was likely the result of poor electrical contact between the particles, either because of blocking due to PVP, or, in the case of the microparticles, because there are fewer points of contact, and those contacts might be covered by an oxide.

### Cu alloy NPs prepared by WE

The morphology and size distribution of Cu alloy NPs were similar to those of Cu NPs. The elemental distribution in the Cu alloy NPs was also carefully investigated using 30Ni sample, because the large amount of Ni made the Ni detection by EDX easy. A fluctuation of Ni content was observed among NPs (Figure S1); however, all of the observed NPs (more than 20 NPs) contained a certain amount of Ni (5.53–49.0 mol%).

**Figure 3:**
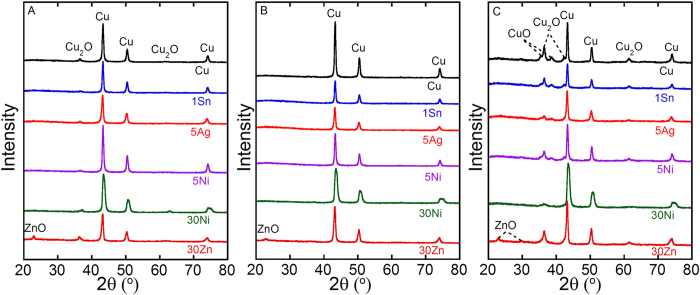
XRD patterns of samples prepared by WE in 200 mM ascorbic acid aqueous solution: (**A**) as-prepared, (**B**) sintered, (**C**) sintered followed by corrosion.

**Figure 4:**
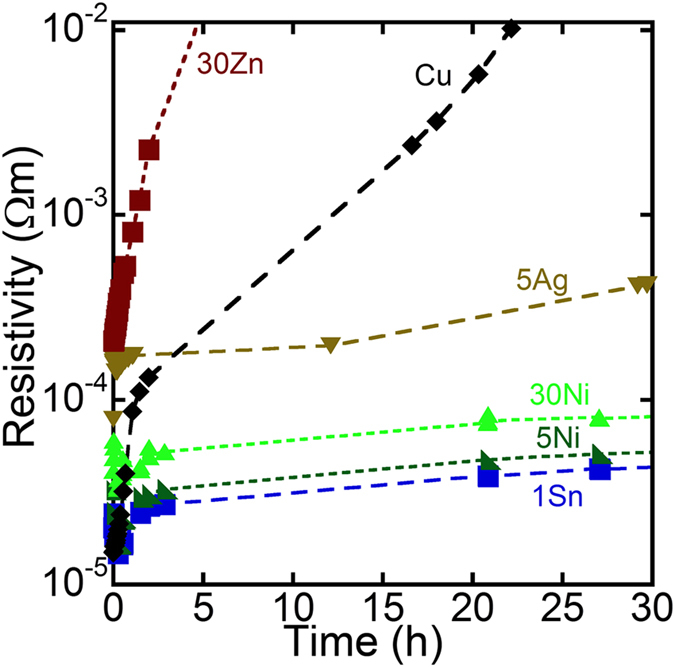
Time-dependent conductivity attenuation during exposure to 85 °C and 85% RH.

Resistivities of conductive lines drawn using Cu and Cu alloy NPs prepared by WE in 200 mM ascorbic acid solution are shown in Table 2. The measured resistivities of the 1Sn, 5Ag, 5Ni, and 30Ni Cu alloy NPs before and after sintering were similar to those of Cu NPs made with 200 mM ascorbic acid, with the lowest alloy content 1Sn exhibiting the best performance. This result makes sense given that the conductivity of Cu alloys generally increases with the concentration of Cu. However, the resistivity of the 30Zn (brass) composition was a couple orders of magnitude higher than the other compositions. Formation of a ZnO shell on 30Zn NPs was confirmed by XRD and TEM analysis (Figure S2), which is consistent with the much higher resistivity of 30Zn than the other pre-sintered samples. The sintering removed Cu_2_O peaks completely from XRD patterns of all samples, but ZnO peaks in 30Zn remained after sintering (Figs 3A and 4B).

Figure 4 shows how the resistivity of lines made from NPs consisting of different alloys change under conditions which accelerate the corrosion of the metal (85 °C and 85% RH). The resistivities measured in this experiment were not consistent with the results shown in Table 2. This was because different pieces of apparatus were used for these two experiments; the detailed conditions are described in the Methods section later. The resistivity of the 30Zn sample and pure Cu sample increased rapidly under these conditions. In contrast, the increase in the resistivity of the 1Sn, 5Ag, 5Ni, and 30Ni lines were relatively slow. After 24 hrs at 85 °C and 85% RH, 4.3, 0.54, 13 and 11% of the initial conductivity was retained for the 1Sn, 5Ag, 5Ni and 30 Ni compositions, respectively. In contrast, only 0.017 and 0.0035% of the initial conductivity was retained for Cu and 30Zn, respectively. The XRD patterns of these samples after the corrosion test revealed the formation of Cu_2_O and CuO in all samples (Fig. 3C), suggesting that the resistivity increase is due to oxidation of Cu. The XRD peak intensities of copper oxides of Cu and 30Zn were stronger than those of the other samples (Fig. 3C). This would explain that more oxides were formed in Cu and 30Zn. In order to eliminate a possibility that a large amount of amorphous oxide was formed in each sample, the energy-dispersive X-ray spectroscopy (EDX) was performed. The results were consistent with the XRD data, i.e., less oxygen was detected by EDX for 1Sn, 5Ag, 5Ni, and 30 Ni compared to Cu and 30Zn (Table S1).

**Figure 5:**
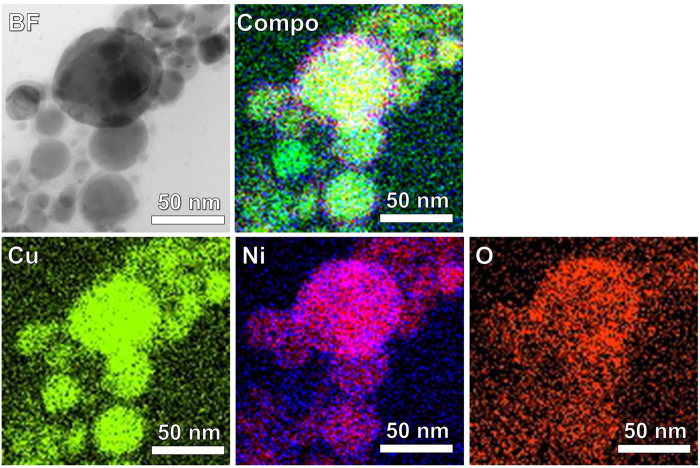
STEM image (BF, bright field) of 30Ni after corrosion testing for 50 hrs. The corresponding EDX mappings for Cu, Ni and O are also shown. The Compo image is composed of overlapped Cu, Ni, and O mappings.

TEM images were obtained for Cu and 30Ni NPs before and after corrosion testing at 85 °C, 85% RH for 1 hr and 50 hrs (Figure S3). Spherical NPs were initially observed for each sample. After 1 hr, a thin oxide layer was formed on each sample. The Cu NPs were completely oxidized after 50 hrs. In contrast, the 30Ni NPs were protected by the passivating oxide on the surface of the NPs, and retained their spherical shape after 50 hrs. EDX measurements were further carried out to confirm the formation of passive oxide layer on the 30Ni NPs after corrosion testing for 50 hrs (Fig. 5). The oxide layer was clearly observed on the surface of 30 Ni NPs in the corresponding scanning-mode TEM (STEM) image (BF, bright field), and the oxide layer contained much larger amounts of Ni and O than the metal core that contains more Cu (see and compare the elemental mappings of Cu, Ni and O, and also the corresponding composed image (Compo)). Since an alloying of Cu with Ni or a coating of Ni on Cu has been employed to improve the oxidation resistance of Cu by forming Ni-containing compact passive oxide layer^43^,^44^, the improved oxidation resistance of 30Ni NPs in this work would be reasonable. Moreover, although clear elemental EDX mappings from the other alloy NPs could not be taken because of the low content of the secondary metals, the similar phenomenon can be expected for the improved oxidation resistance in 1Sn, 5Ag, and 5Ni.

With further improvement of their electrical conductivity and corrosion resistance, Cu alloy NPs prepared by WE could be used for the production of affordable conductive inks for printed electronics^45^,^46^,^47^,^48^,^49^,^50^,^51^. The reproducibility of this method would become sufficient once the corrosion resistance of NPs was further improved. The ability of the conductive inks to be deposited by more advanced printing techniques such as inkjet and screen printings should be evaluated in the near future.

## Conclusion

This article demonstrated that WE of metal wires in an aqueous ascorbic acid solution was a simple and environmentally friendly approach to the production of NPs consisting of Cu and Cu alloys for conductive inks. Cu and Cu alloy NPs produced by WE exhibited electrical resistivities as low as 5 × 10^−5^ Ω m after drying in air at room temperature, and 1.6 × 10^−6^ Ω m after sintering in H_2_. Cu NPs alloyed with 1% Sn, 5% Ag, 5% Ni and 30% Ni had electrical conductivities similar to Cu, but remained conductive after 24 hrs at 85 °C and 85% RH. It is worth mentioning that the best resistivity achieved in this work was still 3000 time (after drying) and 100 times (after sintering) higher than bulk Cu value. Nevertheless, we hope this work motivates additional study of combining WE with chemical modification of the explosion medium to control the composition and surface chemistry of NPs produced by WE.

## Methods

### Source of Wires

Macroscopic wires of Cu (0.079 or 0.4 mM (diameter), Arcor electronics, USA), Ag (0.0254 mM, Superpure chemicals, USA), Ni (0.25 mm, Sigma-Aldrich, USA), tinned Cu (99Cu-1Sn, 0.4 mm, Arcor electronics, USA), and brass (70Cu-30Zn, 0.4 mm, Malin, USA) were cut into 60 mm segments to match the distance between electrodes in a stainless-steel sample chamber (IMNano, Korea). The 1Sn and 30Zn NPs were prepared by exploding wires with compositions of 99Cu-1Sn and 70Cu-30Zn. The 5Ag, 5Ni, and 30Ni NPs were prepared by exploding two separate wires that were twisted together: 1 Cu wire (60 × 0.4 mm) and 13 Ag wires (60 × 0.0254 mm) for 5Ag; 1 Cu wire (60 × 0.4 mm) and 1 Ni wire (8.4 × 0.25 mm) for 5Ni; and 23 Cu wires (60 × 0.079 mm) and 1 Ni wire (60 × 0.25 mm) for 30Ni.

### Preparing Metal NPs by WE

**Figure 6:**
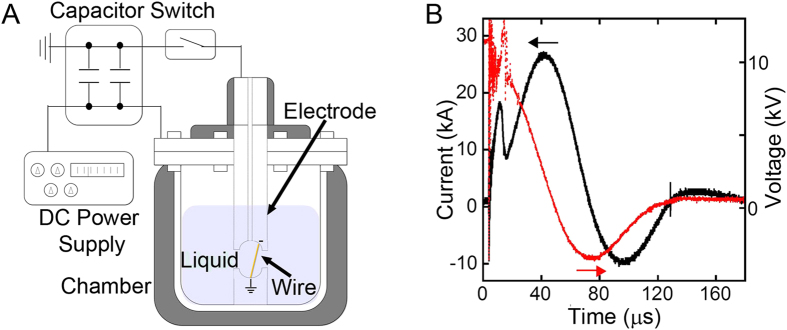
(**A**) Illustration of the wire exploder. (**B**) Dynamics of the voltage and current between the electrodes during WE of Cu in 200 mM ascorbic acid aqueous solution.

Figure 6A shows a scheme of the wire exploder for preparation of metal NPs. Two 20 μF capacitors (NOC20M20CS, Condenser products, USA) connected in series were charged with a high-voltage direct current power supply (Series ER, Glassman high voltage, USA) with an appropriate voltage for each metal wire. The voltage was chosen to generate 5 times more energy than that required for vaporization of the wire 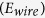, which is calculated as follows:


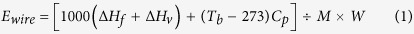


where 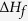 and 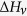 are heat of fusion and vaporization, *T*_*b*_ is boiling temperature, *C*_*p*_ is heat capacity, *M* is the atomic weight of the metal, and *W* is the mass of the wire.

The chamber was filled with 550 ml of deionized water or an aqueous solution of ascorbic acid (>99%, Alfa Aesar, USA) before placing the wire between the electrodes through the nozzle of the chamber (Fig. 6A). Current was passed from the capacitor through the wire by closing the spark gap switch, which was connected with coaxial cables to the positive and negative electrodes of the capacitors. The voltage and current profiles during WE were measured using a digitized oscilloscope (DSO-5200A USB, Hantek, China) connected to a high-voltage probe (P6015A, Tektronix, USA) and a current transformer (Model 3-0.002, Stangenes Industries, USA). As shown in Fig. 6B, the voltage and current profile during WE of Cu features a sharp current rise and drop caused by Joule heating and subsequent explosion. A secondary current rise was caused by the inductive oscillation that results from plasma formation in the vaporized metal^25^,^38^.

After each explosion, a new wire was inserted through the chamber nozzle. This procedure was repeated 5–10 times in order to obtain a sufficient amount of NPs for conductivity tests. The obtained NPs were concentrated by centrifugation of the solution at 1500 rpm, followed by removal of the transparent supernatant, to obtain suspension with approximately 40% NPs by volume. Since the stability of the NP dispersion is relatively low, the centrifugation and replacement of the supernatant should be quickly carried out after the explosion of the wires.

### Characterization

Conductive lines were drawn by drop-casting of 15 μl of solution containing 40 vol% NPs in a Scotch tape template with a 2 × 15 mm hole (see Figure S4). After the solvent was completely evaporated, some lines were sintered at 200 °C in H_2_ for 30 min. The thicknesses of the lines were measured using a profilometer (Dektak 150, Bruker, USA), and the resistivity was calculated from the thicknesses and resistances obtained with a four-point probe (SP4, Lucas signatone, USA). XRD measurements were performed with an X-ray diffractometer (X’pert pro MRD HR, Panalytical, UK). SEM and TEM observations were carried out using scanning (XL30, FEI, USA) and transmission (Tecnai G2 twin, FEI, USA, JEM-2100F, JEOL, Japan) electron microscopes, respectively. Since the sintered samples were baked together and difficult to scrape off, the as-prepared samples were first placed on TEM grids and then sintered. EDX analyses were done using an energy-dispersive X-ray spectroscope (2300T, JEOL, Japan) equipped with the JEM-2100F. The attenuation of the conductivity of the sintered lines under 85 °C and 85% relative humidity (RH) was recorded with a multimeter (87 V true RMS, Fluke, Canada), with the probes in the humidity controlled oven (TestEquity 200H, USA) which contained the samples. Since the multimeter was attached to thin and long stainless steel wires in order to connect to the samples inside the oven, resistivities lower than 1.0  × 10^−5^ Ω m could not be measured.

## Additional Information

**How to cite this article**: Kawamura, G. *et al.* Production of Oxidation-Resistant Cu-Based Nanoparticles by Wire Explosion. *Sci. Rep.* **5**, 18333; doi: 10.1038/srep18333 (2015).

## Supplementary Material

Supplementary Information

## Figures and Tables

**Table 1:** Resistivity of lines drawn with Cu NPs prepared by WE in 4 different solutions.

Wire	Solution	Resistivity (before sintering)	Resistivity (after sintering)
Cu	H_2_O	∞ Ω m	∞ Ω m
Cu	20 mM ascorbic acid	1.61 × 10^−2^ Ω m	2.38 × 10^−5^ Ω m
Cu	200 mM ascorbic acid	8.25 × 10^−5^ Ω m	1.72 × 10^−6^ Ω m
Cu	200 mM ascorbic acid + PVP	5.60 × 10^−4^ Ω m	5.23 × 10^−6^ Ω m

**Table 2:** Resistivity of lines drawn using Cu and Cu alloy NPs.

Sample	Before sintering	After sintering	After corrosion test
Cu	8.25 × 10^−5^ Ω m	1.72 × 10^−6^ Ω m	1.02 × 10^−2^ Ω m
1Sn	3.49 × 10^−4^ Ω m	1.64 × 10^−6^ Ω m	3.85 × 10^−5^ Ω m
5Ag	5.25 × 10^−5^ Ω m	2.21 × 10^−6^ Ω m	4.13 × 10^−4^ Ω m
5Ni	1.14 × 10^−4^ Ω m	5.99 × 10^−6^ Ω m	4.74 × 10^−5^ Ω m
30Ni	9.09 × 10^−3^ Ω m	8.57 × 10^−6^ Ω m	7.67 × 10^−5^ Ω m
30Zn	4.76 × 10^0^ Ω m	3.78 × 10^−4^ Ω m	1.08 × 10 Ω m

The NPs were prepared by WE in 200 mM ascorbic acid solution.
