# Supplementary material for: Production of Oxidation-Resistant Cu-Based Nanoparticles by Wire Explosion
Source: Sci Rep. 2015 Dec 16;5:18333. doi: 10.1038/srep18333 (PMC4680879; doi:10.1038/srep18333)
Supplement: Supplementary Information [file srep18333-s1.pdf]

Supporting Information

Production of Oxidation-Resistant Cu-Based Nanoparticles by Wire Explosion

Go Kawamura[1,2,*], Samuel Alvarez[1], Ian E. Stewart[1], Matthew Catenacci[1], Zuofeng Chen[1,3], and Yoon-Cheol Ha[1,4]

[1] Duke University, Department of Chemistry, Durham, North Carolina 27708, United States
[2] Toyohashi University of Technology, Department of Electrical and Electronic Information Engineering, 1-1 Hibarigaoka, Tempaku-cho, Toyohashi, Aichi 441-8580, Japan
[3] Tongji University, Department of Chemistry, Shanghai 200092, PR China
[4] Korea Electrotechnology Research Institute, Creative and Fundamental Research Division, Changwon 642-120, Korea.
* Correspondence and requests for materials should be addressed to G.K. (email: gokawamura@ee.tut.ac.jp)

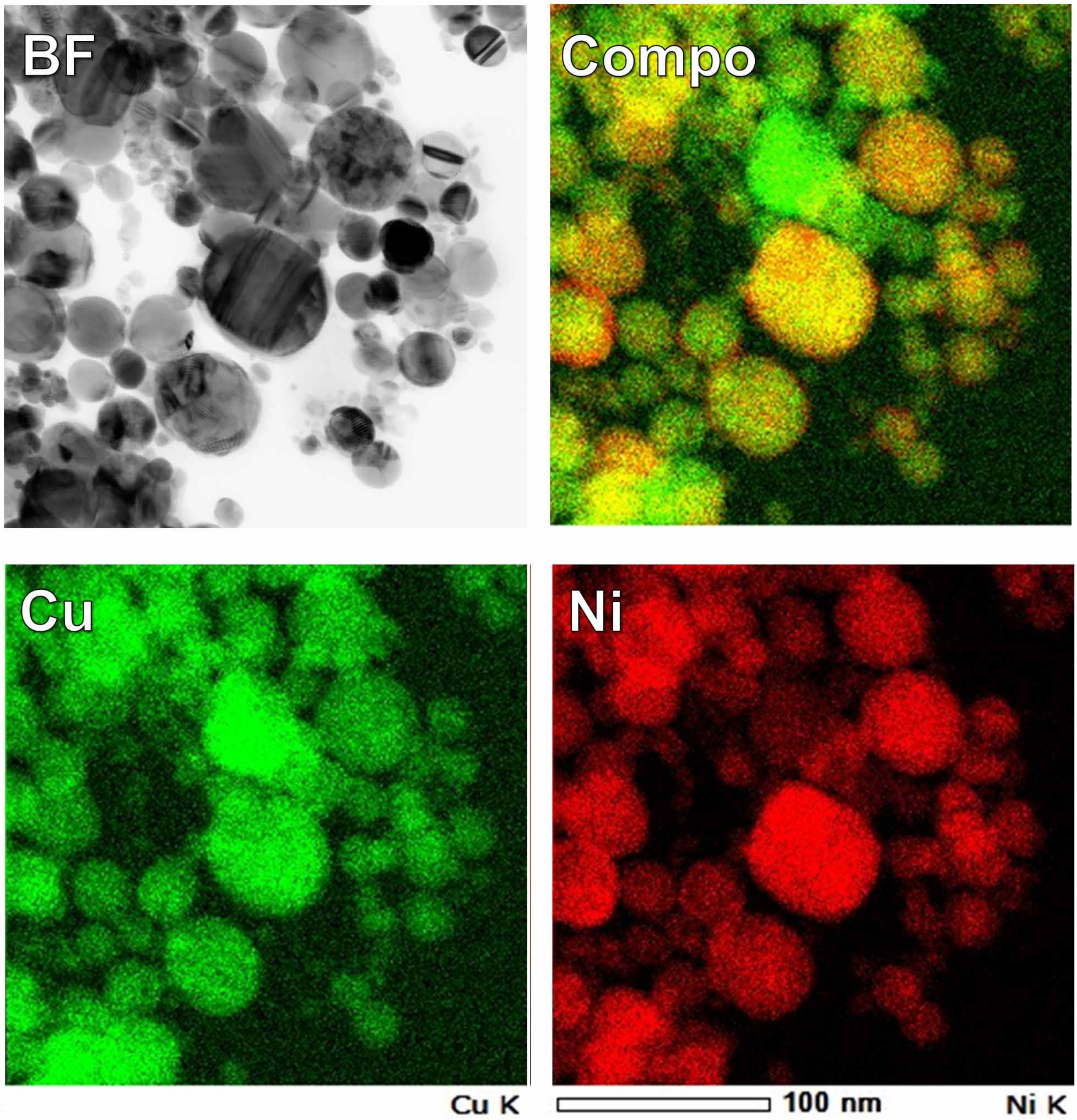

Figure S1. STEM image (bright field, BF) of 30Ni. The corresponding EDX mappings for Cu and Ni elements are also shown. The Compo image is composed of overlapped Cu and Ni mappings.

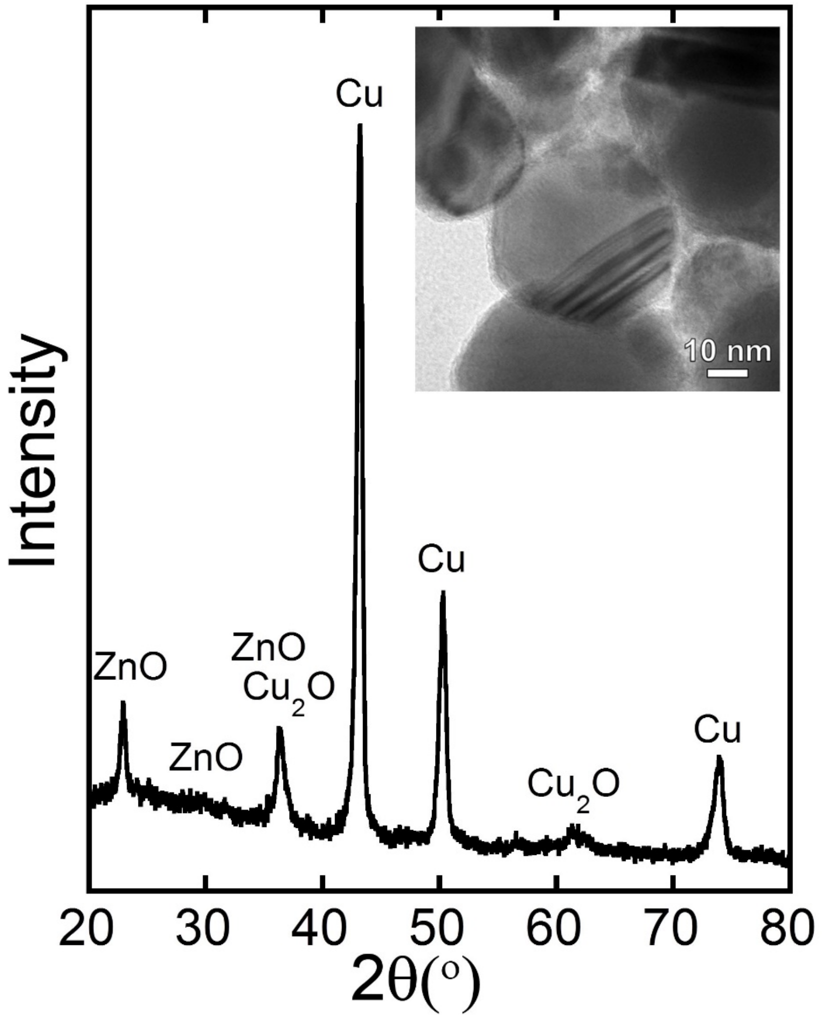

Figure S2. XRD pattern and TEM image of 30Zn NPs prepared by WE in an aqueous solution of 200 mM ascorbic acid.

Table S1. Normalized atomic ratio of oxygen/metal detected by EDX. All samples were measured after corroded at 85 °C, 85% RH for 50 hrs.

**Sample**	**Normalized Oxygen/Metal (at%)**
**Cu**	1.000
**1Sn**	0.373
**5Ag**	0.397
**5Ni**	0.473
**30Ni**	0.330
**30Zn**	1.058

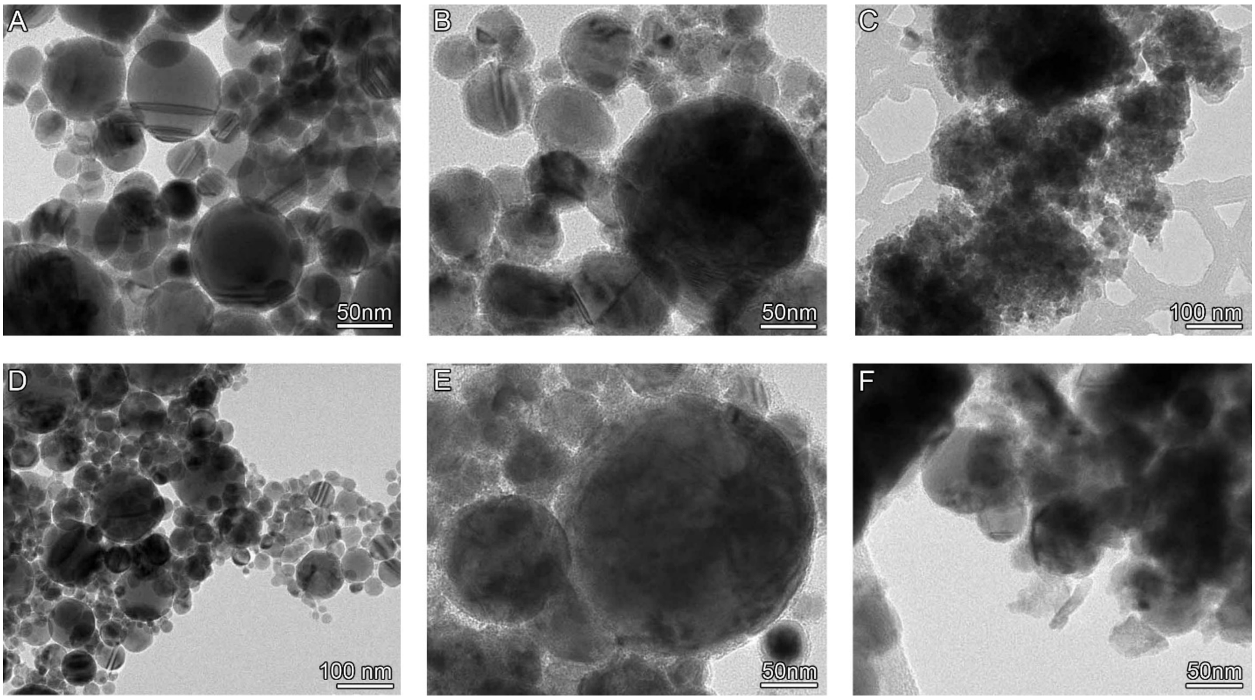

Figure S3. TEM images of (A, B, C) Cu and (D, E, F) 30Ni. (A&D) sintered, (B&E) corroded at 85 °C, 85% RH for 1 hr, (C&F) corroded at 85 °C, 85% RH for 50 hrs.

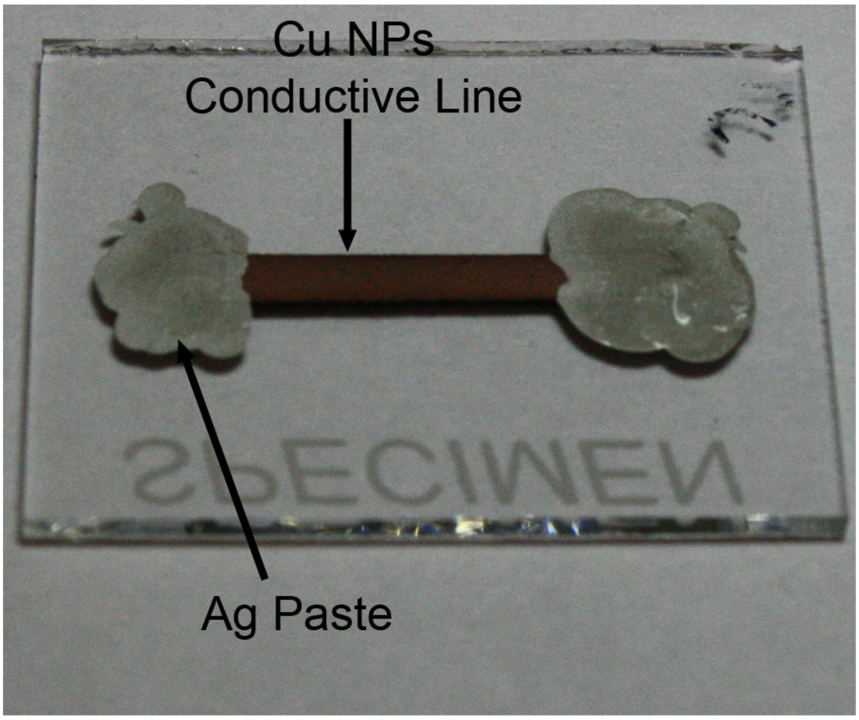

Figure S4. A photograph of a conductive line drawn using Cu NPs prepared by WE in an aqueous solution of 200 mM ascorbic acid. The width, length and thickness of the line were 2, 15 mm and 14.1 μm, respectively.
